# Assessing the Performance of a New Artificial Intelligence–Driven Diagnostic Support Tool Using Medical Board Exam Simulations: Clinical Vignette Study

**DOI:** 10.2196/32507

**Published:** 2021-11-30

**Authors:** Niv Ben-Shabat, Ariel Sloma, Tomer Weizman, David Kiderman, Howard Amital

**Affiliations:** 1 Sackler Faculty of Medicine Tel-Aviv University Tel-Aviv Israel; 2 Department of Medicine ‘B’ Sheba Medical Center Ramat Gan Israel; 3 Kahun Medical Ltd Tel-Aviv Israel; 4 The Rappaport Faculty of Medicine Technion Israel Institute of Technology Haifa Israel; 5 Hadassah Faculty of Medicine The Hebrew University Jerusalem Israel; 6 The Zabludowicz Center for Autoimmune Diseases Sheba Medical Center Ramat Gan Israel

**Keywords:** diagnostic decision support systems, diagnostic support, medical decision-making, medical informatics, artificial intelligence, Kahun, decision support

## Abstract

**Background:**

Diagnostic decision support systems (DDSS) are computer programs aimed to improve health care by supporting clinicians in the process of diagnostic decision-making. Previous studies on DDSS demonstrated their ability to enhance clinicians’ diagnostic skills, prevent diagnostic errors, and reduce hospitalization costs. Despite the potential benefits, their utilization in clinical practice is limited, emphasizing the need for new and improved products.

**Objective:**

The aim of this study was to conduct a preliminary analysis of the diagnostic performance of “Kahun,” a new artificial intelligence-driven diagnostic tool.

**Methods:**

Diagnostic performance was evaluated based on the program’s ability to “solve” clinical cases from the United States Medical Licensing Examination Step 2 Clinical Skills board exam simulations that were drawn from the case banks of 3 leading preparation companies. Each case included 3 expected differential diagnoses. The cases were entered into the Kahun platform by 3 blinded junior physicians. For each case, the presence and the rank of the correct diagnoses within the generated differential diagnoses list were recorded. Each diagnostic performance was measured in two ways: first, as diagnostic sensitivity, and second, as case-specific success rates that represent diagnostic comprehensiveness.

**Results:**

The study included 91 clinical cases with 78 different chief complaints and a mean number of 38 (SD 8) findings for each case. The total number of expected diagnoses was 272, of which 174 were different (some appeared more than once). Of the 272 expected diagnoses, 231 (87.5%; 95% CI 76-99) diagnoses were suggested within the top 20 listed diagnoses, 209 (76.8%; 95% CI 66-87) were suggested within the top 10, and 168 (61.8%; 95% CI 52-71) within the top 5. The median rank of correct diagnoses was 3 (IQR 2-6). Of the 91 expected diagnoses, 62 (68%; 95% CI 59-78) of the cases were suggested within the top 20 listed diagnoses, 44 (48%; 95% CI 38-59) within the top 10, and 24 (26%; 95% CI 17-35) within the top 5. Of the 91 expected diagnoses, in 87 (96%; 95% CI 91-100), at least 2 out of 3 of the cases’ expected diagnoses were suggested within the top 20 listed diagnoses; 78 (86%; 95% CI 79-93) were suggested within the top 10; and 61 (67%; 95% CI 57-77) within the top 5.

**Conclusions:**

The diagnostic support tool evaluated in this study demonstrated good diagnostic accuracy and comprehensiveness; it also had the ability to manage a wide range of clinical findings.

## Introduction

### Background

Diagnostic decision support systems (DDSS) are computer programs that aim to improve healthcare and minimize diagnostic errors by supporting healthcare professionals in the process of diagnostic decision-making [1-3]. These processes, both in general and specifically in medicine, are influenced by cognitive biases [4,5], difficulty estimating pre- or posttest probabilities [6,7], and the experience level of the caregiver [8]. The currently available DDSS vary greatly in terms of knowledge base source and curation, algorithmic complexity, available features, and user interface [9-12]. However, all DDSS generally work by providing diagnostic suggestions based on a patient’s specific data. Previous studies have demonstrated the ability of DDSS to enhance clinicians’ diagnostic skills [2,3,13,14], prevent diagnostic errors [14], and reduce hospitalization costs [15]. However, no effect regarding patient-related outcomes has been reported yet [16,17]. Despite the potential benefits of DDSS and the fact that the first products were introduced decades ago [1,10,11,18], they are not yet widely accepted in the medical community and are not used routinely in clinical practice [17,19]. The factors proposed to be responsible for this state include negative perceptions and biases of practitioners, poor accuracy of the available tools, inherent tendency to prefer sensitivity over specificity, lack of standardized nomenclature, and poor usability and integration into the practitioner’s workflow [16,19-22]. These facts emphasize the need for new products harnessing recent advances in the data science field.

### About the Diagnostic Support System Evaluated

In this study, we evaluated the diagnostic performance of Kahun (Kahun Medical Ltd), a new diagnostic support tool for healthcare practitioners, freely available to use online or as a mobile app. Kahun enables users to input a wide range of findings concerning their patients and, in turn, generates: (1) a differential-diagnoses (DDX) list, ranked according to likelihood; (2) stronger and weaker findings alongside a graph of clinical associations for each suggested diagnosis, all with direct references; and (3) further options for diagnostic workup with evidence-based justifications aimed to refine the DDX, to exclude life-threatening cases, and to reach a definitive diagnosis. A video demonstrating the use of the platform for a standard patient is presented in Multimedia Appendix 1. A series of step-by-step screenshots portraying the different panels and functions of the mobile app is presented in Multimedia Appendix 2.

Kahun’s knowledge base is a structured, scalable, quantitative knowledge graph designed to model both ontological and empirical medical knowledge as they appear in evidence-based literature. To combine aspects of semantic knowledge graphs with empirical and probabilistic relationships, Kahun adopts the techniques of causal graphs and probabilistic graphing models. The platform’s sources of knowledge include core clinical journals and established medical textbooks of internal medicine, as well as ontological poly-hierarchies such as the Systematized Nomenclature of Medicine (SNOMED) and the Logical Observation Identifiers Names and Codes (LOINC) [23]. Each data point is referenced back to the original source, thus enabling the assignment of different weights for each data point according to the strength of evidence of its source. Data from these sources are curated using a model that transforms textual representations into structured interconnections between medical concepts found in the text; these connections point to the specific cohorts and cite the statistical metrics provided by the source. The knowledge base is continuously being updated and growing all the time. It currently contains over 10,000 concepts, alongside 20,000,000 facts and metrics cataloged from over 50,000 referenced sources.

Given a set of findings, the Kahun core algorithm processes information from the structured knowledge base to support the clinical reasoning process. The goal of the algorithm is to highlight all relevant knowledge in the context of a specific patient. Hence, the system is always dealing with a “cohort of one,” meaning a cohort representing patients that match all known attributes of the presented patient. The algorithm can synthesize and transform metrics, where valid (eg, using published sensitivity and likelihood ratio to compute the specificity of a test). Most often, metrics must be estimated despite missing data in the literature. In such cases, the algorithm will estimate probabilities, which are an extension of existing facts and in harmony with other published metrics. The transparency at the heart of the knowledge graph allows all such estimates to be explained, using clinical reasoning, and referenced back to their sources. The Kahun system goes through a constant process of quality assurance, carried out by a combination of medical experts and automated tools. Internal tools provide an on-demand view of knowledge per medical concept (eg, disease, clinical finding, and more), and test reports are produced for the clinical reasoning given patient presentations. Both are tested continuously against data sets of medical cases.

### Objectives

The goal of this study was to test the diagnostic accuracy of Kahun in terms of its ability to suggest the expected diagnosis in a series of cases from the United States Medical Licensing Examination (USMLE) Step 2 Clinical Skills board exam simulations. This is meant to be a preliminary evaluation of the platform, aimed at providing an initial indication regarding its diagnostic capability and general practicality. Further investigations are planned to evaluate its influence on practitioners’ skills and behavior in both simulated and real-life settings, with the end goal of demonstrating its effect on healthcare quality measures and patient-related outcomes.

## Methods

### Case Selection

Cases were extracted from the case banks of 3 leading USMLE board exams preparation companies: UWorld, Amboss, and FirstAid. All cases available for subscribed users were drawn and checked for eligibility. Each case included a summary of the patient’s clinical findings (demographics, medical and family history, medications, habits, symptoms, and signs) and 3 “correct” DDX that are expected to be suggested. The cases were reviewed by 3 physicians, who are registered specialists in emergency medicine, rheumatology, and internal medicine, with at least 5 years of practicing experience. Each case was assigned to a medical discipline based on its chief complaint. Cases from the disciplines of pediatrics, obstetrics, trauma, and psychiatry were excluded if at least 2 reviewers allocated these cases to such groups.

### Procedures and Design

A group of 3 junior physicians, interns in internal medicine from a tertiary hospital in Israel, were recruited to enter the clinical findings of the selected cases into the Kahun platform. To avoid biases and simulate use by an inexperienced user, the selected physicians had no prior experience using Kahun. They were blinded to the correct diagnoses, and the only guidance they received was a short online tutorial video (Multimedia Appendix 1). For each case, the presence and the rank of the correct diagnoses within the generated DDX list were recorded.

### Statistical Analysis

A case was considered successful if Kahun listed the correct diagnosis within the top 5, 10, and 20 places of the generated DDX list, which includes a maximum of the 20 most likely diagnoses. Diagnostic performance was measured in two ways. First, as sensitivity, calculated as the total number of the expected DDX appropriately suggested (within the top 5, 10, and 20 of the listed diagnoses), divided by the total number of the expected diagnoses in all cases. This analysis was further stratified according to organ system. Second, the comprehensiveness of the DDX list was measured and calculated as the number of cases with 1/3, 2/3, and 3/3 of the expected DDX appropriately suggested (within the top 5, 10, and 20 listed diagnoses), divided by the total number of cases. Statistical analysis was performed using the commercial software SPSS (for Windows, version 26.0, IBM Corp). The 95% CIs were calculated assuming binomial distribution.

## Results

### Characteristics of Cases

**Figure 1:**
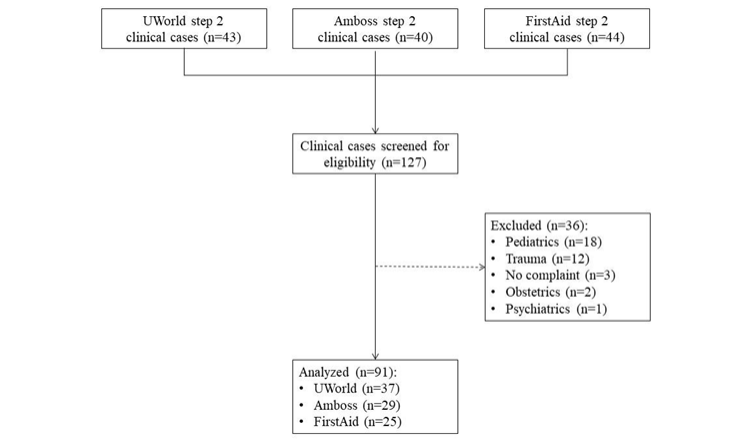
Case selection flow chart.

A total of 127 cases were screened from Amboss (n=40), FirstAid (n=44), and UWorld (n=44); 36 cases were excluded because they were classified as pediatric (n=18), trauma (n=12), obstetric (n=2), and psychiatric (n=1) cases or were routine-checkup cases without a chief complaint (n=3). The remaining 91 cases, Amboss (n=29), FirstAid (n=25), and UWorld (n=37), were analyzed in the study (Figure 1).

Each case was provided with 5 (n=1), 4 (n=1), 3 (n=85), or 2 (n=4) correct diagnoses, resulting in a total of 272 tested diagnoses of which 174 were unique (some diagnoses appeared in more than 1 case). The most common expected diagnosis was hypothyroidism (n=6), followed by adverse drug reaction, pelvic inflammatory disease, hyperthyroidism, pneumonia, and depressive disorder (n=5). The distribution of diagnoses according to organ systems and basic success rates is presented in Table 1. The best diagnostic sensitivity rates were demonstrated for diagnoses related to the digestive system (54/55, 98.2%) and to the genitourinary system (35/36, 97.2%), while the worst were demonstrated for autoimmune or inflammatory diagnoses (8/13, 61.5%). Diagnostic accuracy did not fall below 50% in any category. Overall, 845 different findings (both positive and negative) were entered into Kahun in the test, with a mean number of 39.8 (SD 8) findings for each case.

**Table 1:** Distribution of case diagnoses according to organ system and specific accuracy rates.

Organ system	Accuracy^a^ n/N (%)	95% CI
Cardiovascular	18/21 (85)	71-100
Respiratory	14/18 (77)	59-97
Gastrointestinal	54/55 (98)	95-100
Genitourinary	35/36 (97)	92-100
Infectious	26/30 (86)	75-99
Nervous	22/27 (81)	67-96
Musculoskeletal	4/5 (80)	45-100
Ear-nose-throat	12/16 (75)	54-96
Autoimmune or inflammatory	8/13 (61)	35-88
Endocrine/metabolic/drugs	19/29 (65)	48-83
Psychiatric	17/19 (89)	76-100
Other	2/3 (67)	13-100

^a^Within the top 20 listed diagnoses.

### Diagnostic Sensitivity Rates

Diagnostic sensitivity rates are presented in Table 2. Out of the total 272 expected diagnoses, 231 (87.5%) diagnoses were accurately suggested within the top 20 listed diagnoses (95% CI 76-99), of which 209 (76.8%) were listed within the top 10 (95% CI 66-87), and 168 (61.8%) listed within the top 5 (95% CI 52-71). There was no statistical significance in the difference of sensitivities between the different case sources. The median rank of correct diagnoses was 3 (IQR 2-6).

**Table 2:** Diagnostic sensitivity.

Company name	Correctly suggested diagnoses					
	Within top 5 listed diagnoses		Within top 10 listed diagnoses		Within top 20 listed diagnoses	
	n (%)	95% CI	n (%)	95% CI	n (%)	95% CI
Total (N=272)	168 (61.8)	52-71	209 (76.8)	66-87	238 (87.5)	76-99
Amboss (n=87)	57 (65.5)	49-83	72 (82.8)	64-100	79 (90.8)	71-100
FirstAid (n=76)	43 (56.6)	40-73	56 (73.7)	54-93	61 (80.3)	60-100
UWorld (n=109)	68 (62.4)	48-77	81 (74.3)	58-90	98 (89.9)	72-100

### Diagnostic Comprehensiveness

Case-specific success rates are presented in Table 3. In 62 (68%) out of 91 cases (95% CI 59-78), all of the cases’ expected diagnoses were suggested within the top 20 listed diagnoses; in 44 (48%; 95% CI 38-59), they were listed within the top 10 diagnoses; and in 24 (26%; 95% CI 17-35), within the top 5 diagnoses. In 87 (96%) out of 91 cases (95% CI 91-100), at least 2 out of 3 of the cases’ expected diagnoses were suggested within the top 20 listed diagnoses; in 78 (86%; 95% CI 79-93) within the top 10 listed diagnoses; and in 61 (67%; 95% CI 57-77) within the top 5 listed diagnoses.

**Table 3:** Case-specific success rates.

Top diagnoses	Rate of correctly suggested diagnoses per case (n=91)					
	3/3^a^		≥2/3^b^		≥1/3^c^	
	Cases, n (%)	95% CI	Cases, n (%)	95% CI	Cases, n (%)	95% CI
Within top 5 listed diagnoses	24 (26)	17-35	61 (67)	57-77	84 (92)	87-98
Within top 10 listed diagnoses	44 (48)	38-59	78 (86)	79-93	88 (97)	93-100
Within top 20 listed diagnoses	62 (68)	59-78	87 (96)	91-100	90 (99)	97-100

^a^Including cases with 2/2, 4/4, and 5/5 correct diagnoses.

^b^Including a case with 4/5 correct diagnoses.

^c^Including cases with 1/2 and 2/4 correct diagnoses.

## Discussion

### Principal Results

In this study, we evaluated the diagnostic performance of Kahun, a new open-access DDSS, based on its ability to suggest the expected diagnoses in simulated board exam cases. Overall, Kahun demonstrated good diagnostic sensitivity and comprehensiveness in managing these cases. Moreover, the system demonstrated its ability to manage a wide range of patient-related findings and to reach a wide range of accurate diagnoses from different fields of medicine.

### Comparison to Previous Studies

The general literature addressing computer-assisted diagnosis is vast. However, when we narrow the scope to commercially available systems that adhere to the definition of DDSS (as established by Bond et al [24]) and those that are targeted for general practice rather than a specific field or condition, only a handful of original studies regarding diagnostic accuracy remain [1,12,24,25]. Similar to our study, all of these studies used a structured clinical case model to evaluate diagnostic systems. Of the studies we reviewed, 3 used cases from different case banks [12,24,25], while 1 used structured cases based on real patients [1]. Unlike our study, all of these [1,12,24,25] defined accuracy as the retrieval rate of a single “gold standard” diagnosis in the top 20 or 30 differential diagnoses generated by the tested tool. None of the studies [1,12,24,25] reported the mean rank of correct diagnoses or the number of findings the system was able to include, except for the study by Graber et al [25] on ISABEL (Isabel Healthcare), which used 3 to 6 key findings for each case. Regarding diagnostic sensitivity, a recent comprehensive metanalysis [26], covering 36 original studies, reported a pooled sensitivity of 70% (95% CI 63-77) overall, and 68% (95% CI 61-74) in studies with stronger methodological quality ratings. The highest accuracy rate was observed for ISABEL, which demonstrated a pooled sensitivity of 89% (95% CI 83-94) with a high heterogeneity between studies [26]. Importantly, the studies in which ISABEL demonstrated the highest accuracy rates defined success as the tool’s ability to output the correct diagnosis in a DDX list containing the 30 most likely diagnoses, as opposed to the 20 diagnoses in our study [25]. A recent study [12], comparing Doknosis, DXplain (Massachusetts General Hospital), and ISABEL, analyzed diagnostic accuracy on a data set including cases from the UWorld case bank, which was also used in our study. In this analysis, the best sensitivity rate observed was 47%. Given these findings, it is safe to assume that the diagnostic sensitivity observed in our study falls in the upper range of what was previously demonstrated by the existing systems. Clearly, no direct comparison between the products could be made in our study.

### Strengths

In this study, we used structured clinical cases that simulate the USMLE Step 2 board exams to evaluate a new diagnostic support tool. These cases have the advantage of being principal cases, which are frequently encountered in primary care and emergency department settings. Moreover, they are designated for the level of junior physicians and medical students, who are populations that were demonstrated to benefit the most from using DDSS [3]. An additional advantage was the fact that each case had 3 “correct” diagnoses rather than a single final diagnosis. This more accurately reflects the true nature of these systems: to serve as valuable resources in the hands of the physician by providing reliable and reasoned case-specific diagnostic and workup suggestions, rather than serving as a “Greek oracle” predicting the correct diagnosis [3,13]. This approach also enabled us to assess the comprehensiveness [1,3,13] of the DDX quality. The cases were entered into the platform by first-time users, which increased the platform’s external validity by allowing an extrapolation of the results to those of an “average” user; it also enabled the study to reflect on the instinctive nature of the diagnostic system. This procedure was performed while the subjects were blinded to the correct diagnoses, thus reducing the chance of response bias.

### Limitations

Our study has several limitations. First, it was designed to assess the accuracy of Kahun in an ideal environment, which does not reflect the stressful and time-limiting working environment of a junior clinician in the primary care clinic, emergency department, or internal medicine department settings. Moreover, the patient summaries used in this study were already somewhat processed and do not account for the clinician’s judgment regarding the relevancy of certain findings or the ability to produce and interpret findings from a physical examination. Another shortcoming for this type of comparison is that it measures the accuracy of the diagnostic tool itself, rather than its ability to augment the user’s informed decision-making, which is perhaps a more valuable measure of performance [1,3,13]. For these reasons, caution needs to be taken when extrapolating the results to performance in an actual clinical setting. The clinical cases selected in this study were based on the USMLE board exams, which, although diverse, are less representative of the rare or unique cases usually depicted in case-report studies. Furthermore, they do not include laboratory and imaging findings and, therefore, do not measure the ability of Kahun to handle these findings. Finally, regarding the platform itself, Kahun is currently not set up to manage patients in pediatrics, trauma, obstetrics, and psychiatry settings. Therefore, we were forced to exclude these cases from the analysis. Nevertheless, it is important to note that Kahun was able to generate DDX from these fields with similar accuracy rates.

### Conclusions

Kahun is a new diagnostic tool that demonstrates an acceptable level of diagnostic accuracy and comprehensiveness. Further studies are warranted to evaluate its contribution to the physician’s decision-making process, to the quality of healthcare, and to the clinical outcomes of the patients, including direct comparison to other DDSS.

## Appendix

Multimedia Appendix 1 Tutorial video demonstrating a standard patient’s run in Kahun's platform.

Multimedia Appendix 2 A series of screenshots portraying panels and functions of the mobile app.

## Abbreviations

DDSS: diagnostic decision support system
DDX: differential diagnoses
LOINC: Logical Observation Identifiers Names and Codes
SNOMED: Systematized Nomenclature of Medicine
USMLE: United States Medical Licensing Examination
