# Supplementary figures and images for: Assessing the Performance of a New Artificial Intelligence–Driven Diagnostic Support Tool Using Medical Board Exam Simulations: Clinical Vignette Study
Source: JMIR Med Inform. 2021 Nov 30;9(11):e32507. doi: 10.2196/32507 (PMC8672291; doi:10.2196/32507)

## Slide 1
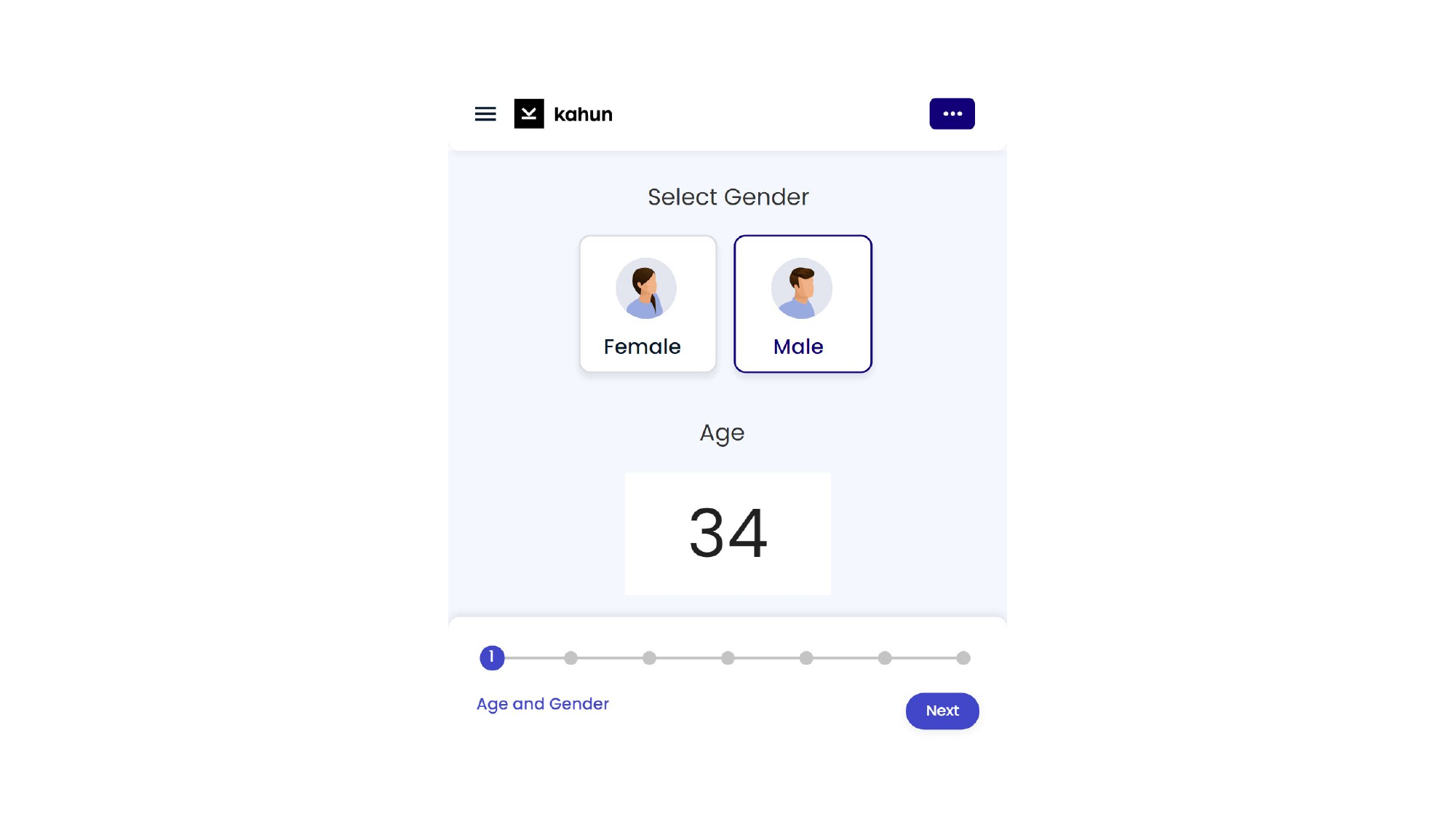

## Slide 2
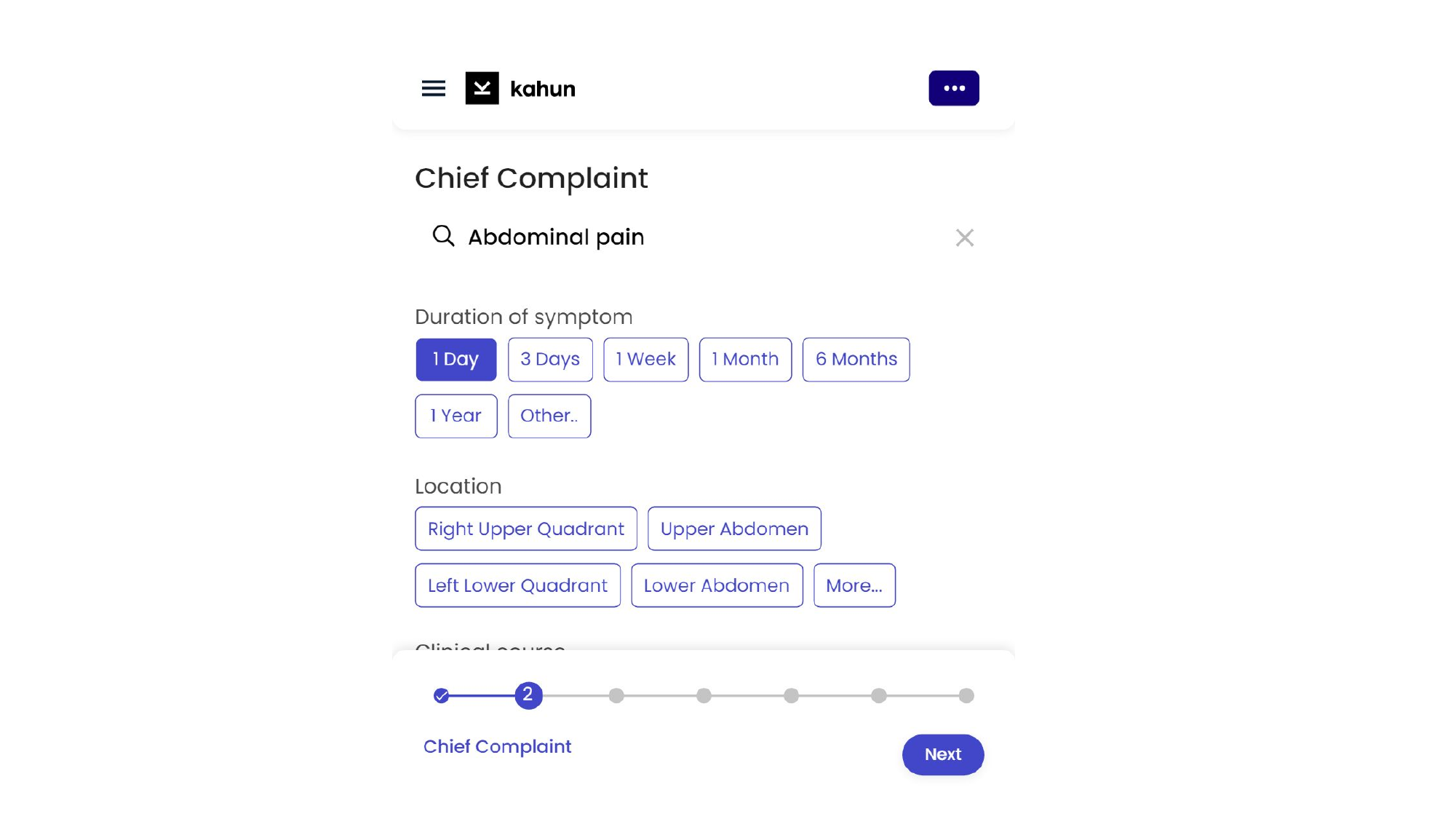

## Slide 3
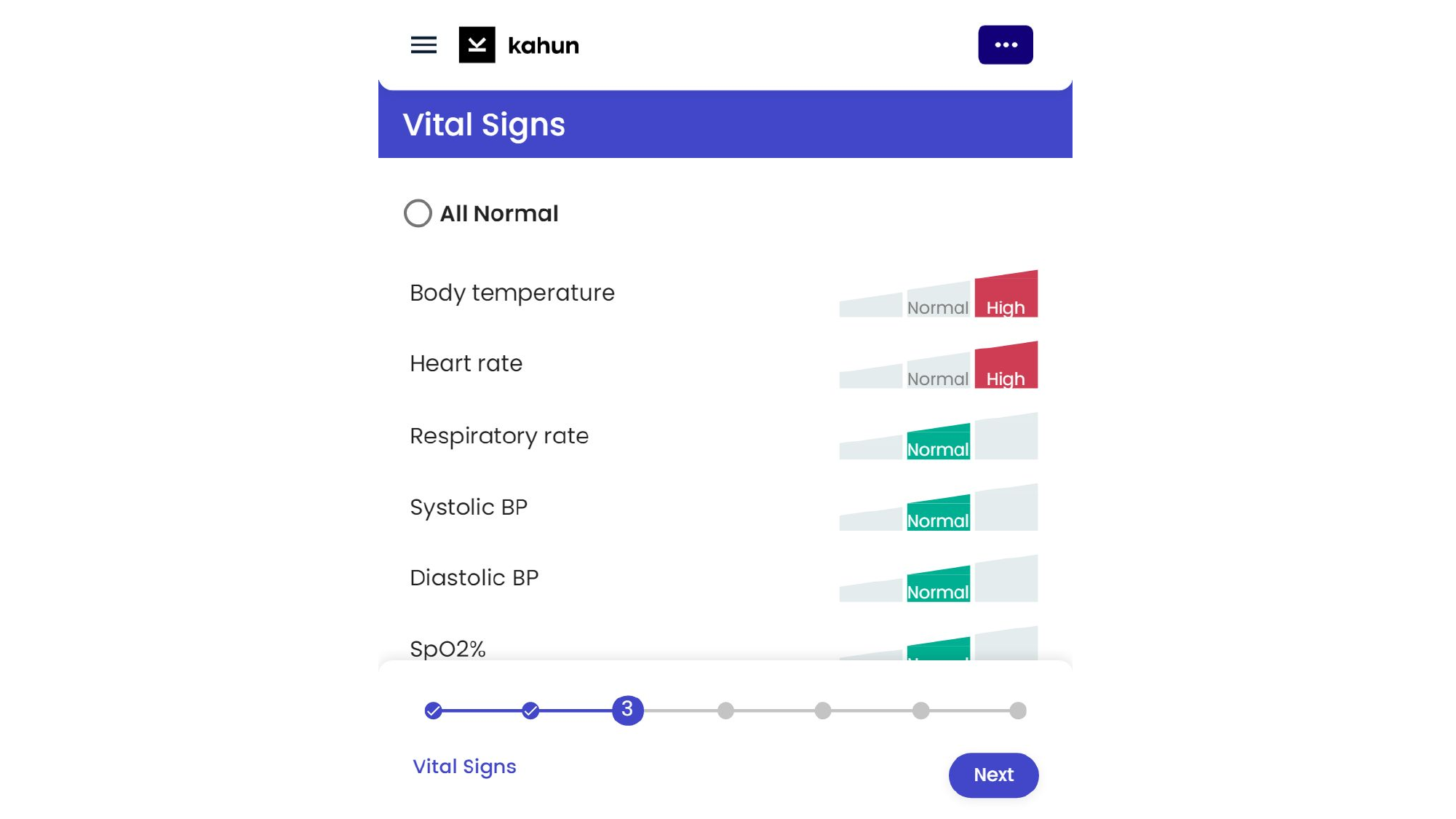

## Slide 4
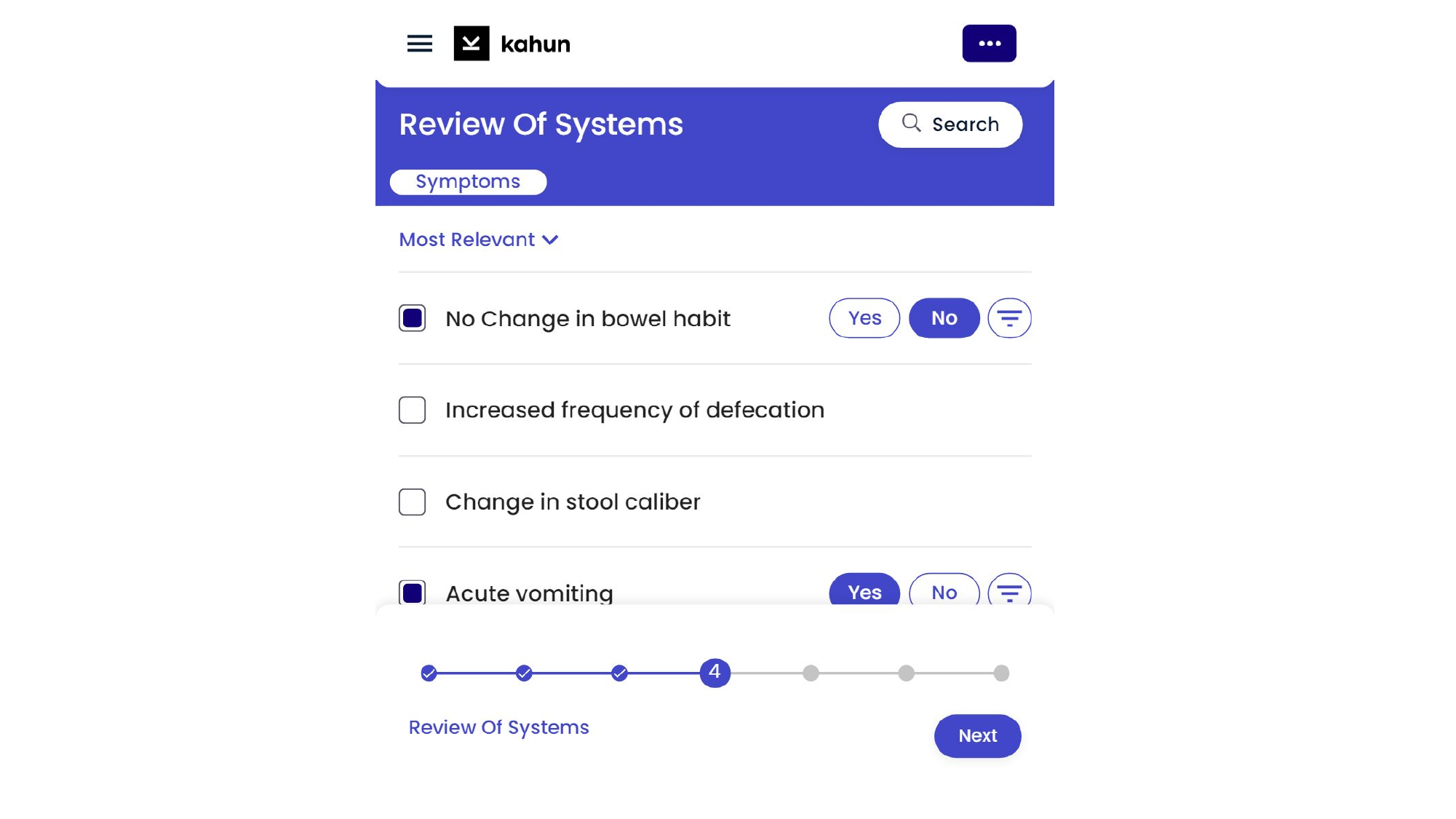

## Slide 5
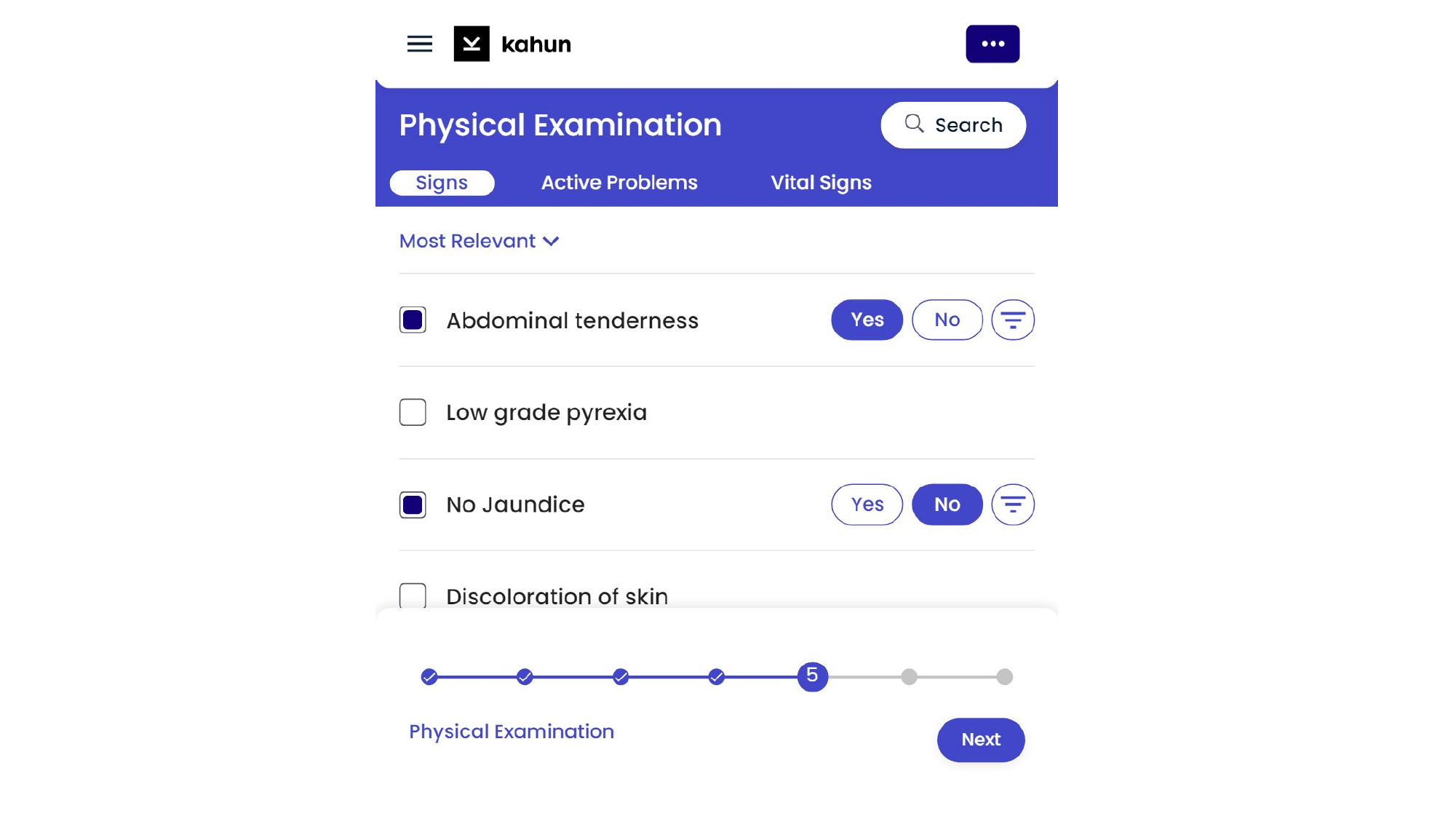

## Slide 6
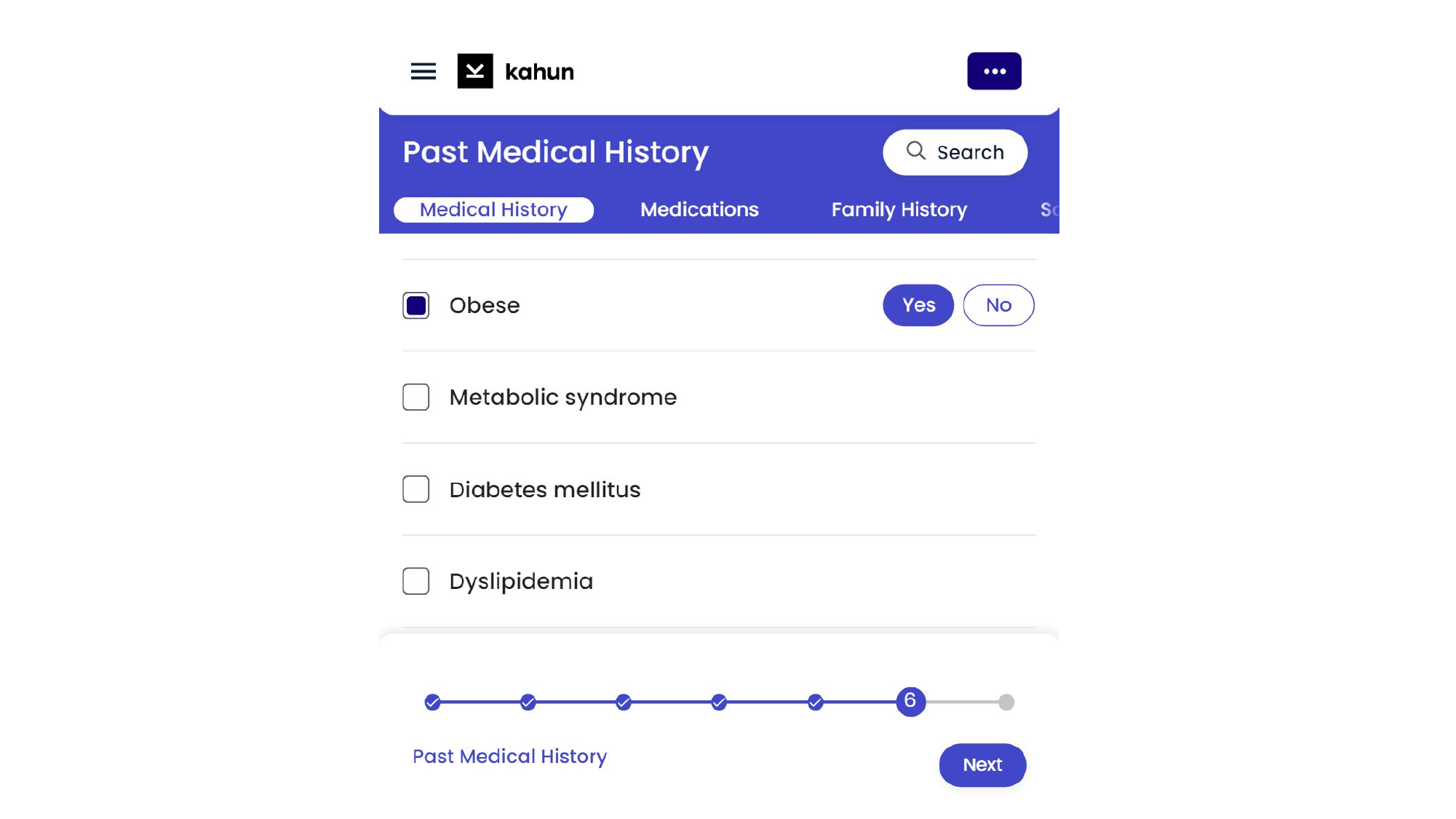

## Slide 7
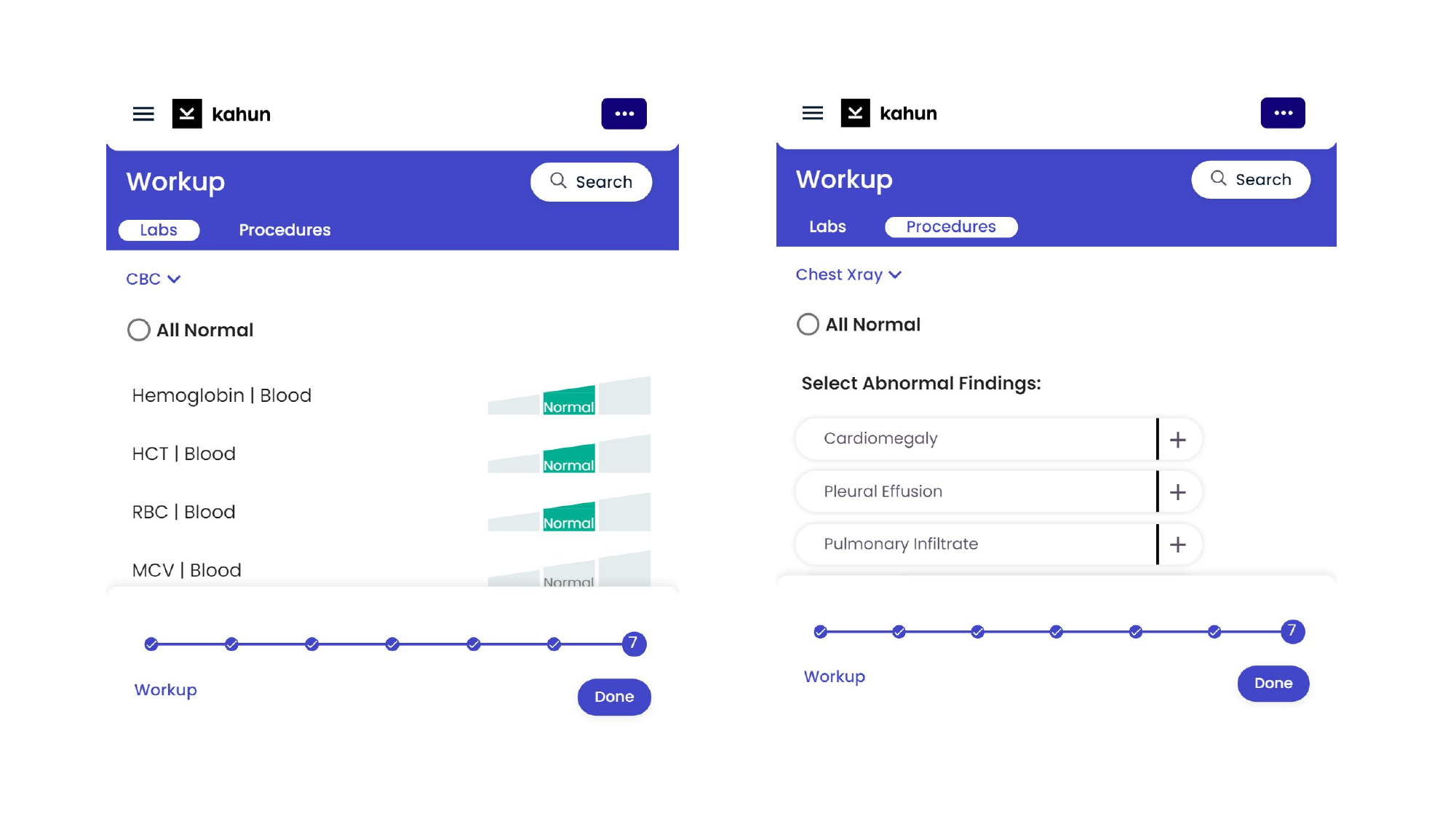

## Slide 8
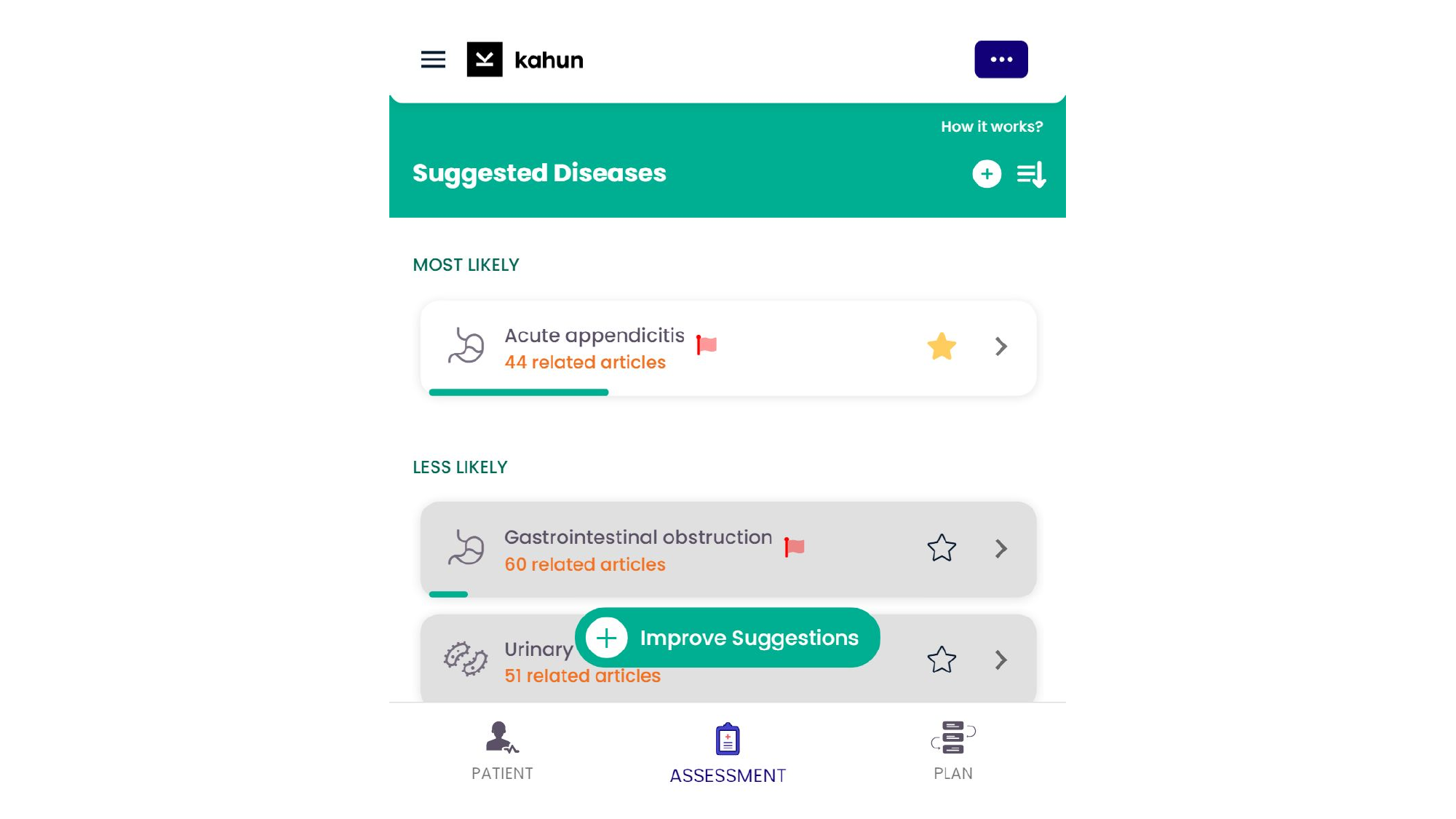

## Slide 9
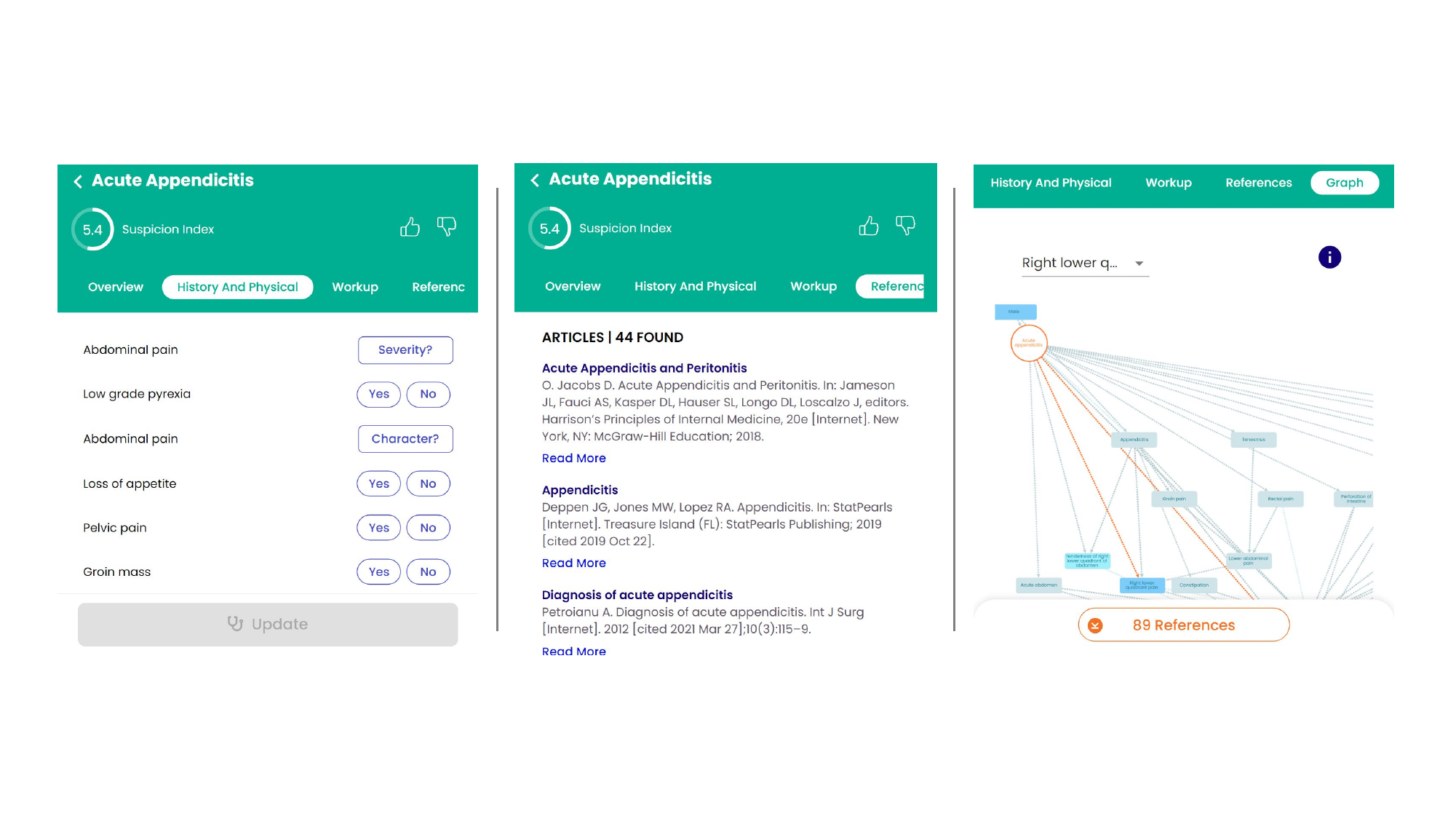

## Slide 10
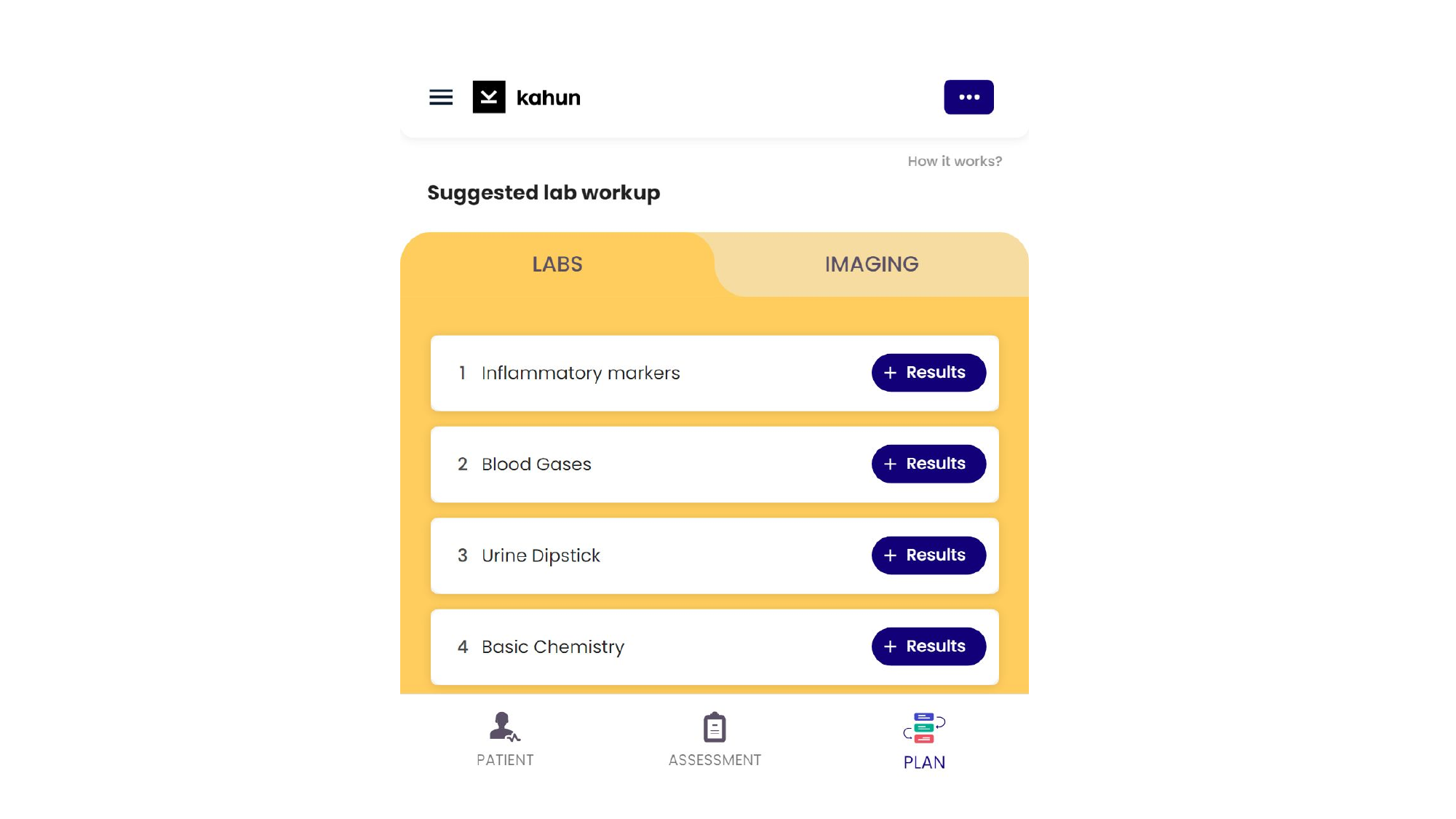

Supplement: Multimedia Appendix 2 [file medinform_v9i11e32507_app2.pptx]
